# Obstetric ultrasound scanning by local health workers in a refugee camp on the Thai–Burmese border

**DOI:** 10.1002/uog.7350

**Published:** 2009-10

**Authors:** M J Rijken, S J Lee, M E Boel, A T Papageorghiou, G H A Visser, S L M Dwell, S H Kennedy, P Singhasivanon, N J White, F Nosten, R McGready

**Affiliations:** * Shoklo Malaria Research Unit (SMRU)Mae Sot, Thailand; ‡ Mahidol–Oxford Tropical Medicine Research Unit (MORU), Mahidol UniversityBangkok, Thailand; † Department of Obstetrics, University Medical CenterUtrecht, The Netherlands; § Centre for Clinical Vaccinology and Tropical Medicine, Churchill HospitalOxford, UK; ¶ Nuffield Department of Obstetrics and Gynaecology, University of OxfordOxford, UK

**Keywords:** accuracy, developing country, fetal biometry, reproducibility, ultrasound

## Abstract

**Objectives:**

Ultrasound examination of the fetus is a powerful tool for assessing gestational age and detecting obstetric problems but is rarely available in developing countries. The aim of this study was to assess the intraobserver and interobserver agreement of fetal biometry by locally trained health workers in a refugee camp on the Thai–Burmese border.

**Methods:**

One expatriate doctor and four local health workers participated in the study, which included examinations performed on every fifth pregnant woman with a singleton pregnancy between 16 and 40 weeks' gestation, and who had undergone an early dating ultrasound scan, attending the antenatal clinic in Maela refugee camp. At each examination, two examiners independently measured biparietal diameter (BPD), head circumference (HC), abdominal circumference (AC) and femur length (FL), with one of the examiners obtaining duplicate measurements of each parameter. Intraobserver measurement error was assessed using the intraclass correlation coefficient (ICC) and interobserver error was assessed by the Bland and Altman 95% limits of agreement method.

**Results:**

A total of 4188 ultrasound measurements (12 per woman) were obtained in 349 pregnancies at a median gestational age of 27 (range, 16–40) weeks in 2008. The ICC for BPD, HC, AC and FL was greater than 0.99 for all four trainees and the doctor (range, 0.996–0.998). For gestational ages between 18 and 24 weeks, interobserver 95% limits of agreement corresponding to differences in estimated gestational age of less than ± 1 week were calculated for BPD, HC, AC and FL. Measurements by local health workers showed high levels of agreement with those of the expatriate doctor.

**Conclusions:**

Locally trained health workers working in a well organized unit with ongoing quality control can obtain accurate fetal biometry measurements for gestational age estimation. This experience suggests that training of local health workers in developing countries is possible and could allow effective use of obstetric ultrasound imaging. Copyright © 2009 ISUOG. Published by John Wiley & Sons, Ltd.

## Introduction

Ultrasound examination of the fetus is a powerful tool for assessing gestational age, detecting multiple pregnancy and intrauterine growth restriction, and determining placental location^1^–^5^. Since the 1990s, almost every pregnant woman in developed countries has had access to between one and four routine scans during uncomplicated pregnancies^6^. However, in most developing countries antenatal ultrasound services are non-existent or inadequate. Those that are available are usually limited to tertiary centers or private hospitals in urban regions^7^–^9^.

A lack of qualified sonographers and a shortage of ultrasound machines, most likely due to their high cost and maintenance difficulties, have been barriers to the implementation of routine ultrasound examination in many antenatal clinics in resource-poor settings. Recently, assistant medical officers, clinical officers, midwives or local radiographers have been identified as potential sonographers^7^, ^10^, ^11^. Given that ultrasound imaging has no value if the ultrasonographer is inadequately trained or inexperienced, recent efforts have concentrated on training^8^. Some African countries have reported promising results from starting ultrasound teaching programs^10^ but, as more developing countries introduce such programs, studies to ensure the quality and consistency of locally trained sonographers will be required.

At the Shoklo Malaria Research Unit (SMRU) local health workers (schooled until 16 years of age) have been trained in basic ultrasound imaging since 2001. They have performed approximately 3000 obstetric ultrasound scans per year in Maela refugee camp over the past 5 years. The aim of this study was to assess the intraobserver and interobserver agreement of fetal biometric measurements performed by these health workers.

## Methods

The SMRU is located on the Thai–Burmese border and has studied the epidemiology, prevention and treatment of malaria in pregnancy since 1986. It has five established clinics, one of which is based in refugee camp Maela, where Karen people (a minority group in Burma) are the principal inhabitants. In all its clinics the SMRU runs a program of antenatal care (ANC) to detect and treat all parasitemic episodes during pregnancy through weekly malaria screening in order to prevent maternal death^12^. Since the inception of this ANC program, all pregnant women have been encouraged to attend as early as possible in pregnancy. At the first visit (usually between 8 and 13 weeks' gestation), ultrasound imaging is used to determine viability, detect multiple pregnancy and estimate gestational age. A second scan is performed at 18–24 weeks to confirm gestation, viability and placental position. In women who do not have an early scan, gestational age assessment is based on fetal biometry scans between 18 and 24 weeks' gestation, or using the Dubowitz gestational age examination at birth if no such scan is available^13^.

**Figure 1:**
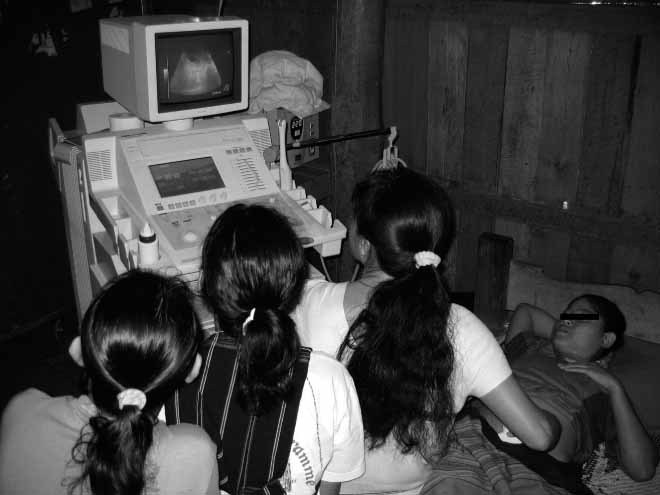
Photograph showing training in the ultrasound room at Maela refugee camp, 2008.

The SMRU introduced ultrasound examination for gestational age assessment owing to the low proportion of women who could reliably provide the date of their last menstrual period (LMP). In the past 3 years only 31% (994/3184) of women in Maela refugee camp provided a reliable LMP. When the ultrasound department in the antenatal clinic of the Maela refugee camp opened in 2001, one of the coauthors (S.L.M.D.), a local Karen health worker who was already skilled in Dubowitz assessment of gestational age, was trained in ultrasound gestational age assessment. A 3-month course of practical and theoretical training in obstetric ultrasound imaging was then developed (Figure 1) for newly employed staff, all of whom were chosen at interview on the basis of motivation, willingness to learn and proficiency in English. The course was based on World Health Organization (WHO) guidelines and British Medical Ultrasound Society (BMUS) recommendations^14^, ^15^. During the next 3 months all scans were verified by a senior sonographer. Only when the head of the department was satisfied with each person's scanning skills and written examination results were they permitted to scan alone.

As part of a larger fetal growth study, quality control evaluation (interobserver and intraobserver variability) was performed between four local sonographers and one expatriate doctor (M.J.R.), certified and experienced in obstetric ultrasound scanning. The Mahidol–Bangkok and Oxford University ethics committees approved both the main and quality control studies.

Every fifth pregnant woman attending the ANC was invited to participate in the study if she had an early (8–13-week) dating scan at the SMRU ANC, a singleton pregnancy, and a gestational age of between 16 and 40 weeks. A maximum of 15 women for each gestational week were invited. After obtaining written informed consent, an abdominal ultrasound examination was performed. At each examination, two examiners independently measured biparietal diameter (BPD), head circumference (HC), abdominal circumference (AC) and femur length (FL) in millimeters. Each image was acquired according to BMUS guidelines, ensuring that the image filled at least a third of the monitor screen. The machine automatically calculated the gestational age (weeks and days) from each measurement using Hadlock's charts^16^. Each examiner was blinded to his own results and the results of the other examiner. All measurements were obtained twice by one examiner (Examiner 1) to assess intraobserver variability, and once by another examiner (Examiner 2) to assess interobserver variability, resulting in 12 measurements per woman (i.e. three sets of four measurements).

All scans were performed using a Toshiba Powervision 7000 machine (Toshiba, Tokyo, Japan) with a 3.75-MHz convex probe, which was donated by the University of Utrecht, The Netherlands. Owing to electrical surges in the refugee camp, a voltage stabilizer was used to operate the ultrasound scanner.

### Statistical analysis

The extent to which measurements agree between two sonographers is limited by the amount of variation in repeated measurements made on the same subject by the same individual. This measurement error was assessed using the intraclass correlation coefficient (ICC), with a value of 1 (possible range 0 to 1) indicating no measurement error^17^. However, as the ICC will be artificially inflated owing to the large range of gestational ages included, summary measures (mean, minimum and maximum differences and SD) for each sonographer are also reported.

The agreement between the mean of the two measurements made by Examiner 1 and the measurement made by Examiner 2 was then estimated, using the 95% limits of agreement method proposed by Bland and Altman^18^, ^19^. Data were initially plotted, with a line of equality, to gauge the degree of agreement between measurements. All points would lie on the line of equality if the two examiners reported exactly the same measurements. The assumptions that the SD of repeated measures was not related to the magnitude of the ultrasound measurements and that the differences between the measurements followed a normal distribution were then checked visually using scatter plots and histograms, respectively. If the assumptions were not met, calculations were carried out on log-transformed values and the antilog was taken to obtain limits of agreement that could be related to the original scale of measurement^18^, ^20^. As gestational age assessment in clinical practice normally occurs between 18 and 24 weeks, interobserver variation was calculated for this subgroup of measurements, as well as for the entire dataset.

Biometry measurements in the second trimester have an accuracy of ± 1 week in estimating gestational age, whereas the accuracy decreases to ± 2 weeks in the third trimester^21^–^25^. A difference in measurements (millimeters) between the two examiners that corresponded to a difference in gestational age of ± 1 week or less was considered to be very good agreement^21^–^25^. Clinically acceptable and unacceptable findings were gestational age differences that were ± 1–2 weeks and more than ± 2 weeks, respectively.

We used the mean of the repeated measurements taken by Examiner 1 against the measurements made by Examiner 2 to assess interobserver variation. One could expect the estimate of SD to be smaller (owing to removal of repeated measurement error) by using the mean^18^. When compared with using only one set of measurements from Examiner 1, however, the results differed by less than 1 mm for all parameters, regardless of whether the first or second set of repeated measurements was used (results not shown). Therefore, only the results using the mean of Examiner 1's measurements are reported here, and no adjustments to the SD were made.

All analyses were carried out using STATA/SE, version 9.2 for Windows (StataCorp LP, College Station, Texas, USA).

## Results

Between April and September 2008, 349 pregnant women consented to the ultrasound examination. The median gestational age was 27 (range, 16–40) weeks. It was possible to complete the examination and obtain all 12 measurements in all women, and so a total of 4188 measurements were obtained.

### Education level of local health workers

All four local health workers involved in the obstetric ultrasound course agreed to participate in this quality control study. One had completed 3 years of training as a nurse at a recognized institution in Burma. The others did not have any tertiary education but had completed school to grade 10 (16 years old). At the start of the study, they had a median of 20 (range, 12–62) months of work experience.

### Intraobserver variation

Table 1 shows the summary of the repeated measurements of all examiners. The ICC for all four parameters (BPD, HC, AC, FL) was greater than 0.99 for all four trainees and the doctor (range 0.996–0.998), indicating that almost all of the variation observed was due to differences between patients rather than differences in the repeated measurements taken by an examiner on any one patient.

**Table 1:** Mean, minimum and maximum differences for each pair of measurements obtained by the same locally trained sonographer (A–D) or the doctor

		BPD (mm)		HC (mm)		AC (mm)		FL (mm)	
					
Examiner	*n*	Mean difference (SD)	Min, max	Mean difference (SD)	Min, max	Mean difference (SD)	Min, max	Mean difference (SD)	Min, max
A	157	0.15 (1.62)	− 5.5, 7.7	0.43 (6.52)	− 26, 20	0.01 (8.54)	− 24, 36	− 0.28 (1.56)	− 5.4, 4.3
B	70	− 0.11 (1.05)	− 2.3, 4.9	0.90 (4.86)	− 14, 16	0.51 (5.90)	− 14, 24	0.03 (1.27)	− 3.3, 5.1
C	67	− 0.26 (1.43)	− 3.7, 2.9	− 0.64 (6.62)	− 22, 15	− 0.93 (7.97)	− 33, 14	0.14 (1.59)	− 3.6, 8.7
D	18	0.08 (1.46)	− 2.0, 3.3	− 1.44 (4.51)	− 9, 5	0.39 (10.6)	− 30, 27	0.21 (1.53)	− 2.5, 3.2
Doctor	37	0.05 (1.16)	− 3.2, 3.3	1.27 (4.56)	− 6, 19	0.41 (5.45)	− 13, 12	− 0.02 (0.91)	− 1.9, 2.1

AC, abdominal circumference; BPD, biparietal diameter; FL, femur length; HC, head circumference; max, maximum difference; min, minimum difference; *n*, number of pairs of measurements obtained by each examiner.

### Interobserver variation

**Figure 2:**
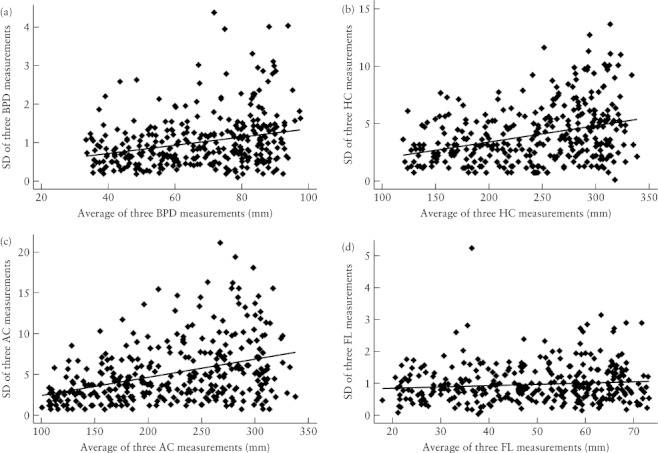
Scatter plots of SD against the average of three measurements for biparietal diameter (BPD) (a), head circumference (HC) (b), abdominal circumference (AC) (c) and femur length (FL) (d) in each fetus. For each graph, the solid line represents the regression line.

**Figure 3:**
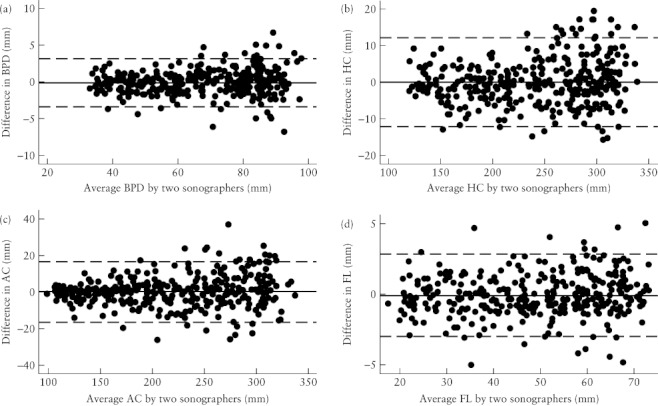
Bland–Altman plots of the interobserver differences in the measurement of biparietal diameter (BPD) (a), head circumference (HC) (b), abdominal circumference (AC) (c) and femur length (FL) (d), showing that the variation increased with the magnitude of the measurements for BPD, HC and AC. For each graph, the solid line represents the mean difference and the dashed lines are the mean difference ± 2 SD.

The agreement between the mean of the two measurements made by Examiner 1 was compared with the single measurement made by Examiner 2 to assess interobserver variation. This was done for the complete dataset (Table 2) as well as for the subset of measurements obtained for pregnancies at 18–24 weeks (Table 3). The distribution of mean differences was approximately normal for each of the four parameters but the SDs and differences for BPD, HC and AC increased with the magnitude of the measurements (Figures 2 and 3). These parameters were log-transformed for analysis and the back-transformed values used to estimate the ‘V-shaped’ 95% limits of agreement (i.e. the range within which measurements were expected to agree 95% of the time increased as the size of the measurement increased)^18^, ^20^.

**Table 2:** Mean difference in ultrasound measurements obtained by two different examiners on 349 women at 16–40 weeks' gestation

Parameter	*n*	Mean difference (mm (95% CI))
BPD	349	− 0.12 (−0.30 to 0.06)
HC	349	− 0.11 (−0.77 to 0.54)
AC	349	− 0.09 (−0.98 to 0.80)
FL	349	− 0.13 (−0.28 to 0.03)

AC, abdominal circumference; BPD, biparietal diameter; FL, femur length; HC, head circumference.

**Table 3:** Mean difference and 95% limits of agreement (LOA) by measurement and corresponding estimated gestational age of ultrasound measurements obtained by two different examiners on 90 fetuses at 18–24 weeks' gestation

		Measurement			Gestational age	
			
Parameter	*n*	Mean difference (mm (95% CI))	SD (mm)	95% LOA (mm)	Mean difference (weeks (95% CI))	SD (weeks)
BPD	90	− 0.43 (−0.68 to − 0.17)	1.21	− 2.80 to 1.94	− 0.12 (−0.19 to − 0.04)	0.36
HC	90	− 1.63 (−2.63 to − 0.62)	4.80	− 11.0 to 7.77	− 0.12 (−0.20 to − 0.03)	0.42
AC	90	− 0.59 (−1.77 to 0.60)	5.65	− 11.7 to 10.45	− 0.03 (−0.13 to 0.07)	0.50
FL	90	− 0.35 (−0.64 to − 0.07)	1.37	− 3.03 to 2.33	− 0.11 (−0.21 to 0)	0.50

AC, abdominal circumference; BPD, biparietal diameter; FL, femur length; HC, head circumference.

### Femur length

The only parameter for which the SDs and the differences were constant throughout the range of measurements was FL (Figures 2 and 3). The mean difference between the ultrasound measurements for each fetus obtained by the two different examiners is presented in Table 2. On average, the measurement of femur length by Examiner 1 differed from the measurement made by Examiner 2 by –0.13 mm (95% CI, –0.28 to 0.03 mm) (Table 2). The 95% limits of agreement ranged from − 3.03 to 2.78 mm. This meant that the measurements of Examiner 2 were likely to be within − 3.03 to 2.78 mm of Examiner 1's measurements 95% of the time, a difference that corresponds to a ± 1–1.5-week variation in gestational age estimation.

### Biparietal diameter, head circumference and abdominal circumference

To account for the increase in variation that occurred with the increase in magnitude of the measurements, the values for BPD, HC and AC were log-transformed to calculate the limits of agreement. These were then back-transformed so they could be related to the original scale of measurement^18^, ^20^. For HC and BPD, 95% of measurements by Examiner 2 could be expected to be 0.95–1.05 times the measurement by Examiner 1. This meant that the measurements by Examiner 2 could differ by 5% above or below that of Examiner 1. If the HC measurement by Examiner 1 was 116 mm (minimum HC), corresponding to a gestational age of 15 + 3 weeks, we would expect the measurement by Examiner 2 to be within ± 5.8 mm 95% of the time. This corresponds to an acceptable variation in estimated gestational age of ± 3 days (15 + 0 and 15 + 6 weeks). The variation increased with the size of the measurement. Therefore, if the HC measurement obtained by Examiner 1 was 284 mm, we would expect the measurement by Examiner 2 to be within ± 14.2 mm 95% of the time. An HC of 284 mm corresponds to a gestational age of 30 + 0 weeks, and ± 14.2 mm to a possible difference of 1.5 weeks, which was considered as just clinically acceptable. Similarly, for a measurement of 344 mm (the maximum HC, corresponding to 39 + 3 weeks) we would expect a possible difference of 4 weeks, larger than clinically acceptable.

Performing similar calculations for BPD, there was a variation of ± 0.5 weeks for the minimum BPD and ± 2.5 weeks for the maximum BPD.

The largest variation between examiners was seen for AC measurements. Those made by Examiner 2 differed from the measurements of Examiner 1 by ± 7% 95% of the time.

### Gestational age assessment in clinical practice

**Figure 4:**
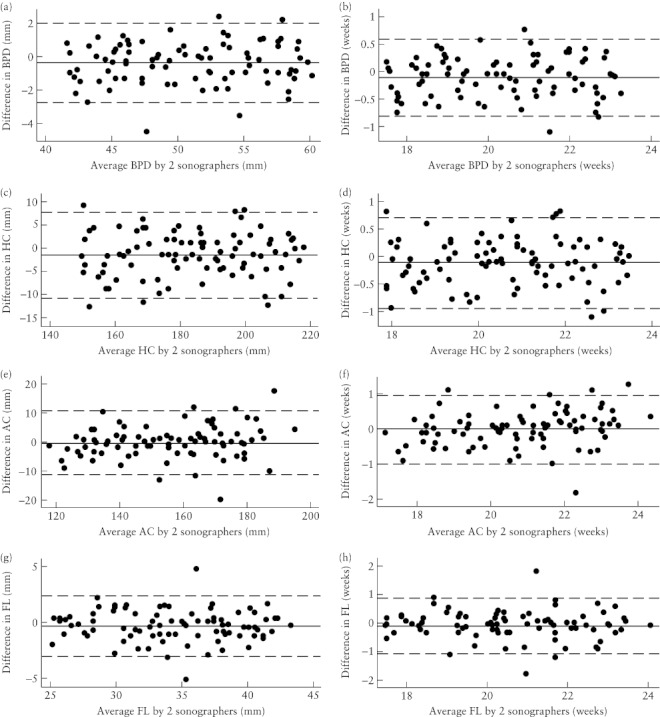
Bland–Altman plots of the interobserver differences in the measurement of biparietal diameter (BPD) (a,b), head circumference (HC) (c,d), abdominal circumference (AC) (e,f) and femur length (FL) (g,h) at 18–24 weeks' gestation, expressed as the measurement itself (a,c,e,g) and the corresponding estimated gestational age (b,d,f,h). For each graph, the solid line represents the mean difference and the dashed lines are the mean difference ± 2 SD (see Table 3).

Between 18 and 24 weeks (when biometry scans are used to assess gestational age if no first-trimester crown–rump length measurement is available), the variation in measurements was constant throughout the range of measurements (Figure 4); mean differences (95% CI) and limits of agreement of this subgroup were therefore calculated without log-transformation (Table 3).

The largest mean difference was for HC measurements (Table 3 and Figure 4), indicating that the measurements by Examiner 2 differed from those made by the first examiner by 1.63 mm (95% CI, 0.62–2.63 mm). The 95% limits of agreement indicated that the measurements made by Examiner 2 could be expected to be within 11.0 mm lower to 7.8 mm higher than the measurements made by Examiner 1, 95% of the time (Table 3). This corresponds to a possible difference in estimation of gestational age of less than ± 1 week. Similarly, differences of less than ± 1 week were estimated for BPD, AC and FL.

### Comparison between the doctor and local trainees

**Figure 5:**
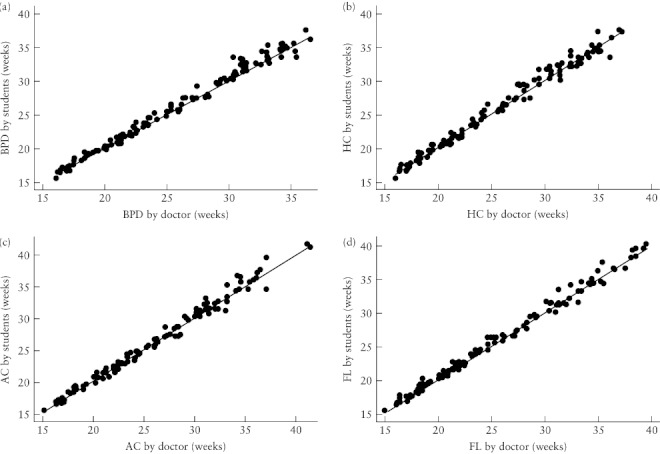
Scatter plots of fetal biometry measurements (*n* = 124) made by the students against those made by the doctor for biparietal diameter (BPD) (a), head circumference (HC) (b), abdominal circumference (AC) (c) and femur length (FL) (d), with the line of equality shown for each.

The expatriate doctor took at least one set of measurements on 124 women. Scatter plots between his measurements and those made by the trained health workers showed that all points were tightly clustered around the line of equality for all four parameters (Figure 5), indicating a high degree of agreement in ultrasound use by both teacher and students.

## Discussion

In this study we found that local health workers can be trained to use ultrasound imaging reliably and consistently to assess gestational age. The intraobserver variation (ICC all > 0.99) demonstrated that measurements are made consistently by the same sonographer. For gestational ages between 18 and 24 weeks, there was a difference of less than 1 week in gestational age estimated using the measurements made by different examiners. In addition, when compared with the skill of an experienced doctor, the local trainees demonstrated a high level of agreement in measuring all four parameters. These findings reassured us that the criteria for selecting the trainees were adequate.

In developed countries it has long been established that fetal biometry at between 14 and 22 weeks' gestation can accurately predict gestational age within ± 7 days (± 2 SD)^21^–^25^. The variation in measurements between examiners in this study falls within this period. Our overall findings, therefore, strengthen the argument that obstetric ultrasound imaging can be introduced in developing countries for gestational age assessment, particularly when one considers the unreliability of LMP recall in such settings^13^, ^26^.

It is essential to maintain quality control in any antenatal ultrasound service to ensure that the data obtained are clinically meaningful, e.g. by estimating the accuracy and reproducibility of the fetal biometry measurements taken by sonographers^18^, ^27^–^29^.

In SMRU clinics, quality control is achieved by routinely taking all ultrasound measurements twice to assess intraobserver variability, and by an expatriate doctor qualified in ultrasound imaging annually checking the skills of all sonographers. Reassuringly, therefore, the measurement errors for gestational age estimation in this study were comparable to those obtained by highly experienced sonographers^29^.

Every measurement in clinical science is associated with error and, unsurprisingly, the variation increased as BPD, HC and AC sizes increased. This was not the case for FL, perhaps because the clearly defined landmarks of the FL (two edges of the femur bone, which are not affected by fetal breathing as in AC measurements) might have contributed to reducing the variation between measurements.

Apart from trained health workers, robust ultrasound machines are needed to make obstetric ultrasound imaging available in remote areas. Unfortunately, as observed by Kurjak and Breyer, ‘many developing countries cannot afford to buy good quality ultrasound diagnostic instruments and do not have enough trained specialists who can devote a large fraction of their active time to the science and art of ultrasound diagnosis’^7^. However, ultrasound imaging has become more feasible in developing countries as machines become less expensive and require less servicing^6^, ^10^, ^30^, ^31^.

To solve the problem of the lack of trained sonographers, the WHO has recognized the urgent need to raise education levels in ultrasound scanning in developing nations^32^. Hence, in 1998, it published a report concerning the essentials, principles and standards of training for both physicians and allied health professionals in diagnostic ultrasound imaging^14^. In reality, however, physicians in developing countries are heavily overloaded with work, resulting in inadequate use of available ultrasound machines^7^, ^8^. Thus, other health workers have been identified as potential sonographers^7^, ^10^, ^11^.

Several reports of international ultrasound training programs have been published previously^10^, ^32^–^34^. Some of these were based in district hospitals in developing countries, where local health workers were trained successfully in theoretical and practical scanning skills. In this study, we have shown that candidates with limited or no tertiary education and limited English in a refugee camp can acquire good quality basic ultrasound skills for gestational age estimation with a short training course, a period of on-the-job training and ongoing quality control measures.

To our knowledge, this is the first report on quality assurance of gestational age estimation of locally trained sonographers in a refugee camp. Our results show that adequately trained health workers, working in a well organized unit with ongoing quality control, can obtain accurate fetal biometry measurements, whether the scan is performed by the same sonographer or by different sonographers. Given the importance of gestational age assessment in obstetric management, we recommend that ultrasound machines are made available and that ultrasound training is provided for local health workers in developing countries or resource-poor settings.
